# Compression and Strain Predictive Models in Non-Structural Recycled Concretes Made from Construction and Demolition Wastes

**DOI:** 10.3390/ma14123177

**Published:** 2021-06-09

**Authors:** Evelio Teijón-López-Zuazo, Jorge López-Rebollo, Luis Javier Sánchez-Aparicio, Roberto Garcia-Martín, Diego Gonzalez-Aguilera

**Affiliations:** 1 Department of Construction and Agronomy, Campus Viriato, Higher Polytechnic School of Zamora, University of Salamanca, Avenida Requejo, 33, 49022 Zamora, Spain; eteijon@usal.es; 2 Department of Cartographic and Land Engineering, Higher Polytechnic School of Ávila, University of Salamanca, Hornos Caleros, 50, 05003 Ávila, Spain; daguilera@usal.es; 3 Department of Construction and Technology in Architecture (DCTA), Escuela Técnica Superior de Arquitectura de Madrid (ETSAM), Universidad Politécnica de Madrid, Av. Juan de Herrera 4, 28040 Madrid, Spain; lj.sanchez@upm.es; 4 Department of Mechanical Engineering, Campus Viriato, Higher Polytechnic School of Zamora, University of Salamanca, Avenida Requejo, 33, 49022 Zamora, Spain; toles@usal.es

**Keywords:** construction and demolition waste, recycled concrete aggregate, recycled ceramic aggregate, non-structural concrete, 3D digital image correlation, predictive models

## Abstract

This work aims to investigate different predictive models for estimating the unconfined compressive strength and the maximum peak strain of non-structural recycled concretes made up by ceramic and concrete wastes. The extensive experimental campaign carried out during this research includes granulometric analysis, physical and chemical analysis, and compression tests along with the use of the 3D digital image correlation as a method to estimate the maximum peak strain. The results obtained show that it is possible to accurately estimate the unconfined compressive strength for both types of concretes, as well as the maximum peak strain of concretes made up by ceramic waste. The peak strain for mixtures with concrete waste shows lower correlation values.

## 1. Introduction

The construction industry is considered the largest consumer of non-renewable natural resources and it is also an important generator of waste [1]. The high consumption of resources is justified by the high demand for concrete in the sector, which is the most widely used artificial material in the world [2]. Although the raw materials and natural aggregates used for manufacture are abundant resources, their high exploitation and costs derived from their extraction entail a problem that can cause the shortage of this type of material in the medium to long term in many countries where its production is very high [3]. Construction is also considered a “dirty” industry due to the high generation of waste [4], both in its extraction phase and in the demolition of elements that have concluded their useful life. Most of these wastes are deposited in landfills, with the consequent negative visual, landscape and ecological impact that this entails. In this context, effective management is necessary in order to reduce both the amount of resources consumed and the amount of waste generated, taking advantage of its potential as secondary material in accordance with the concepts of sustainable development and circular economy.

One of the main solutions that stimulate the reuse of construction and demolition waste (CDW) is its use in the manufacture of concrete. However, the main problem in the use of this type of aggregate is focused on the high absorption capacity due to the presence of ceramic material and mortar adhered to the surface of the aggregates [5,6], especially in the fine ones [7]. This issue reduces the mechanical performance of the resulting concretes. In this sense, the standards on recycled materials, such as the Spanish EHE [8], are quite demanding both in their physical and chemical properties, hindering the use of these materials for the manufacture of structural concrete due to the high requirements in waste treatment [9]. Consequently, it is necessary to establish alternative applications in which the concrete resulting from the use of recycled aggregates does not require high mechanical performance, that is, non-structural concrete (NSC). Multiple studies have been focused on analysing the applicability of this type of concrete, among which are paving blocks [10], kerbstones [11], blocks [12,13] or even prefabricated urban furniture pieces [14].

These new concrete solutions made with recycled aggregates have very different behaviours and one of the main variables that most affects performance is the percentage of replacement of natural aggregates. In this sense, numerous studies have analysed the behaviour according to different mix proportions [11,15,16], suggesting a lower compressive strength for higher percentages of replacement of the natural aggregate, as well as greater strain and a lower modulus of elasticity. However, other studies suggest that the mechanical performance of recycled concrete is similar to that made with natural aggregates, even superior in terms of its compressive strength [17] or tensile performance [18,19].

When the execution of these type of concretes is carried out, it is essential to have tools that allow us to predict their behaviour considering the different mix proportions and curing ages (i.e., hardening curves). However, the high variability of mix proportions based on the substitution of recycled aggregates and their typologies with the consequent diversity of results, increases the difficulty of establishing strength prediction models occuring in conventional concretes [20,21]. Moreover, prediction models that incorporate variables, such as the substitution of natural aggregates for recycled aggregates [22,23], provide a useful design tool and promote the application of sustainable concretes.

Together with the strength values, it is often very useful to know the maximum strain associated with concretes. In this sense, Eurocode 2 [24] provides an expression that allows us to determine the peak strain as a function of the characteristic strength of concrete and that can be used to predict the strain based on the strengths estimated from the curves of hardening. This expression has been adjusted by González-Fonteboa et al. [25] for different substitution percentages of coarse recycled aggregates, but does not take into account the total substitution of coarse and fine aggregates.

The use of CDW as a substitution for aggregates increases the heterogeneity of concrete solutions, showing very different behaviours, if adequate selection and treatment of waste is not carried out [26]. In this sense, the methods generally used to analyse the stress-strain curves and the maximum strain of recycled concrete adapt locally since they are based on point measurements of virtual extensometers [25,27,28]. In practice, concrete failure occurs promptly when the material reaches its maximum strength at the point a fracture begins. This means that the strain, prior to the concrete failure, can be very different if measurements close to the cracks are considered or, on the contrary, the values are taken in very distant areas.

In order to cope with the limitation previously shown, various full-field optical methods have been developed, among which, Digital Image Correlation (DIC) stands out. This method allows us to obtain a full field of displacements and strains through the use of correspondence procedures based on correlations and numerical differentiation algorithms [29]. Thanks to this, DIC has been widely used for the analysis and characterization of various heterogeneous materials, such as wood [30], composites [31] and concrete [32]. In this last field, there are studies that cover both the measurement of strains [33] and the analysis of cracks [34,35], and even the characterization of the influence of aggregate size on drying contractions [36]. The variability of the technique allows carrying out analysis in the plane of cubic specimens using 2D-DIC [34,35] and analysis in non-flat specimens using 3D-DIC [33,36,37], taking into account the variations outside the main plane.

Considering the heterogeneous nature of these materials and the variability of their behaviour, it is worth highlighting the importance of carrying out the DIC approach to analyse the spatial distribution of the displacements and strains suffered throughout the specimen. The 3D-DIC approach allows us to obtain a large amount of data that facilitates statistical analysis and the estimation of properties [32]. In this way, the peak strain can be studied over a large surface of the specimen, thus analysing the failure zone and determining the peak strain more precisely as compared to other specific measurements that may be far from the actual behaviour of the material. As a result of this technique, it is possible to establish a more precise model to predict strains based on the maximum strength of concretes made with recycled aggregates.

As a consequence, this paper aims to progress understanding in the mechanical characterization of non-structural concretes manufactured with construction and demolition waste through generating predictive models of compressive strength and peak strain. For this, the tests of mechanical characterization of concrete with different mix proportions and curing ages will be integrated together with the 3D-DIC approach for the analysis of the strains suffered during the breaking tests. Within the Materials and Methods section, we describe the materials used in the mixture as well as the experimental and numerical strategies adopted. In Section 3, we show the experimental results obtained by the combination of DIC and the mechanical characterization tests. Then, in Section 4, predictive models are defined and discussed. Finally, in the Conclusion (Section 5), we summarize the findings and discuss future studies.

## 2. Materials and Methods

### 2.1. Materials and Mix Proportions

Structural concretes are designed for structures and elements for building or for public works, usually designed for compressive stresses of 25 MPa at 28 days [8]. It is made up by of natural siliceous aggregates, Portland cement and chemical additives such as fluidizers. Within the scope of this study, we focus on evaluating the use of wastes from crushing of this type of concrete in selective demolitions processed at the CDW treatment plant in Calvarrasa de Abajo (Salamanca, Spain) as recycled aggregates for the manufacture of non-structural concrete. More specifically, this study evaluates two potential types of recycled concrete for non-structural applications (borders and sidewalks): (i) a concrete coming from Construction and Demolition Waste from Recycled Concrete (CDWRCon) and; (ii) a concrete made up by the Construction and Demolition Waste from Recycled Ceramic (CDWRCer). In both cases, cement, water and additive were mixed. 

On the one hand, CDWRCon is obtained from concrete, cement mortar and prefabricated concrete parts, including untreated aggregates and natural stone aggregates treated with hydraulic binders and other fractions (content < 0.1%) of floating material, cohesive materials (clay and soil), metals (ferrous and non-ferrous), wood, gypsum and non-floating plastics and rubber. Meanwhile CDWRCer is obtained from concrete, including concrete products, cement mortar, prefabricated concrete parts and ceramic materials, showing a continuous granulometry with concrete and ceramic material aggregates. The CDWRCer components are the same as CDWRCon with the addition of other materials as parts of clay (bricks and tiles), calcium silicate masonry, non-floating aerated concrete and glass.

**Figure 1:**
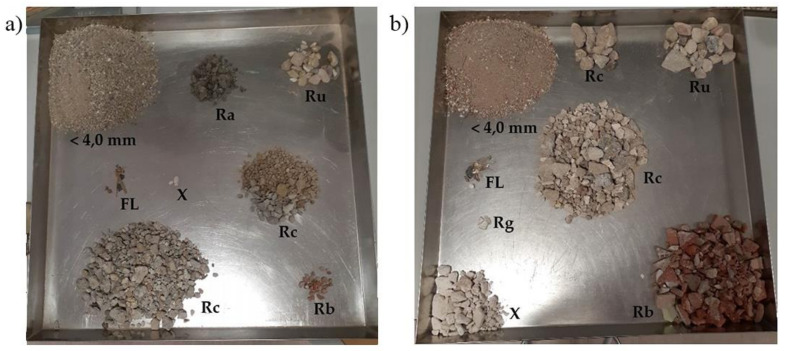
Visual appearance of the aggregates: (**a**) CDWRCon and (**b**) CDWRCer components.

According to the categories established in the UNE-EN 13242 standard [38], the CDWRCon aggregates are classified as *Rc*_80_, *Rcu*_90_, *Rb*_10−_, *Ra*_10−_, *X*_1−_, *FL*_5−_ and the CDWRCer aggregates are classified as *Rc*_40_, *Rcu*_50_, *Rb*_50−_, *Rg*_2−_, *FL*_5−_ (Table 1) (Figure 1), where:

*Rc* = Concrete and mortar (natural aggregates with cement mortar attached).
*Ru* = Unbound aggregates (natural aggregates without cement mortar attached).
*Rb* = Ceramics (bricks, tiles, stoneware and sanitary ware).
*Ra* = Asphalt.
*Rg* = Glass.
*FL* = Floating materials.
X = Other impurities (wood, paper, metals, plastic, etc.).

It is noteworthy that the UNE-EN 13242 [38] standard establishes a maximum of 1% for class *X*, which includes cohesive materials (clay and soil), metals (ferrous and non-ferrous), wood, gypsum, non-floating plastics and rubber. The CDWRCer has an amount of gypsum that represents 5.2% in the general classification of the components, not corresponding to the class established by the standard for this type of material. The gypsum is prejudicial to concrete due to its composition of sulphate and, therefore, it is necessary to consider a sulphate resistant cement.

Table 2 shows the particle size distribution. In CDWRCon, values of uniformity coefficient, *Cu* = 75.0, and curvature coefficient, *Cc* = 2.1, were obtained. The high value of the uniformity coefficient shows the high size variation obtained in the unclassified crushing. The curvature coefficient, 1.0 ≤ *Cc* ≤ 3.0, defines the CDWRCon and the CDWRCer as well-graded and with a low compressibility, a high compactness and correspondingly suitable for use on construction sites.

Additionally, a granulometric study was carried out. In this case it was compared the granulometric curves of both concretes (CDWRCon and CDWRCer) with respect to the Bolomey dosing (reference curve) in accordance to the standard UNE EN 933-1 [39]. This reference curve, which is considered as an improvement of the Fuller law, is adequate for mass concrete (i.e., non-structural concrete), where the resistance is not the determining characteristic.

**Figure 2:**
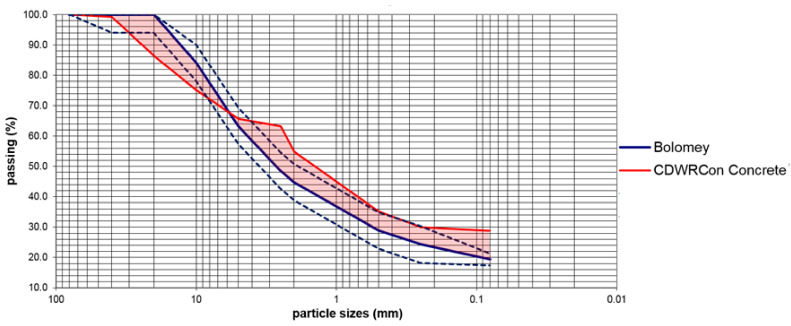
CDWRCon versus Bolomey granulometric curves.

The results of this comparison are shown in Figure 2. It is worth mentioning that the granulometric curve of the CDWRCon concrete includes the cement used as an aggregate (20% of the total volume of aggregates). The Bolomey curve was estimated by considering a wet mix macadam with the following proportions [40]: (i) ±2% for the 0.063 mm; and (ii) ± 6% for the rest. As this research aims to investigate the manufacture of a sustainable concrete, promoting the use of recycled aggregates replacing natural aggregates in concrete, the continuous granulometry obtained in the crushing of the CDWs has been used in the production of the concrete. Thus, better particle size adjustments have been avoided by classifying it into different fractions, as this would be a commercial disadvantage for its implementation in practice. In the CDWRCon the biggest deviations are in the 20 mm sieve with 13.6% above and 14.7% on the 2.5 mm sieve. There is a little standard error of 2.5% below the medium curve. This curve has the typical form of crushing siliceous aggregates, with a deficit of intermediate sizes in the sand fraction, between the #5–2.5 mm sieves. Even with the logical limitations associated with the heterogeneity of the recycled aggregates, the theoretical dosage curve was close to the bottom of the sieve stack for sizes larger than 6 mm in the case of CDWRCon, while the smaller sizes are above the average stack. It can be seen how the areas between the medium spindle and the particle size curve of the CDWRCon are partially compensated, below the medium spindle in the larger sieves and above in the smaller ones (Figure 2).

**Figure 3:**
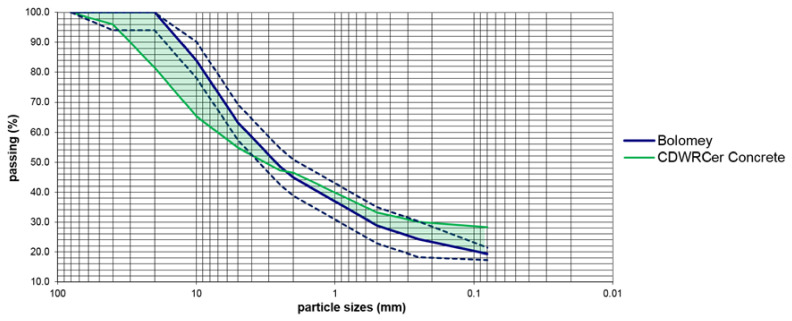
CDWRCer versus Bolomey granulometric curves.

Identically, the granulometric curves of the CDWRCer and Bolomey dosing are shown in Figure 3. In this case, the biggest deviations are in the 10 mm sieve with 18.9% above and 9.0% on the 0.08 mm sieve. There is a small standard error of 3.1% above the medium curve. Even with the logical limitations associated with the heterogeneity of the recycled aggregates, the theoretical dosage curve was close to the bottom of the sieve stack for sizes larger than 2.5 mm in the case of CDWRCon, while the smaller sizes are above the average stack. It can be seen how the areas between the medium spindle and the particle size curve of the CDWRCon are partially compensated, below the medium spindle in the larger sieves and above in the smaller ones.

From the results obtained it is possible to conclude that both groups have the right proportions of sand and gravel to make the necessary concrete for non-structural applications. Table 3 shows the main physical and chemical parameters of CDWRCon and CDWRCer.

where:

*SE*_4_ = Sand Equivalent, UNE-EN 933-8 [41].
*LA* = Los Angeles coefficient, UNE-EN 1097-2 [42].
*AM* = Methylene blue (UNE EN 933-9) [43].
*OM* = Organic Matter content, UNE 103204 [44].
*SS* = Soluble Salt content, UNE 103205 [45].
*WA_f_* = Water Absorption Fine aggregate, UNE-EN 1097-6 [46].
*WA_c_* = Water Absorption Coarse aggregate, UNE-EN 1097-6 [46].
*SO*_3_ = acid soluble sulphate content, UNE-EN 1744-1 [47].
*S* = sulfur compounds total content, UNE-EN 1744-1 [47].
*m_lpc_* = light contaminant content, UNE-EN 1744-1 [47].
*Humus* = light organic contaminant in humus content, UNE-EN 1744-1 [47].

The quality of the fines, expressed as *SE*_4_ sand equivalent, gives a value of 55.9 in CDWRCon. This value is lower than the common values of natural aggregates. The sand equivalent for CDWRCer is even lower than the CDWRCon with a value of 45.3. This difference is motivated by the existence of parts of clays (bricks and tiles), as well as calcium silicate masonry elements that reduce the sand lecture and *SE*_4_ value. Since non-structural recycled concretes (NSRC) are not subjected to any specific exposure class, the aggregates shall be accepted if satisfy the requirement AM ≤ 0.3 f/100.

Fragmentation resistance offers an *LA* coefficient of 43.0 for CDWRCon. The resistance to fragmentation in CDWRCer, with *LA* = 52.0, is lower than CDWRCon. This difference is motivated by the presence of bricks, tiles and calcium silicate masonry. So, the coarse aggregates have an abrasion resistance between 40 and 55, being possible to make non-structural concrete with a minimum characteristic strength of 15 N/mm^2^.

The organic matter test provided a 0.14% value in CDWRCon. The content of soluble salts dissolved in distilled water for CDWRCon was 1.1%. The CDW has a high-water absorption coefficient, higher than natural aggregates. In the CDWRCon, the high absorption was associated with the porosity of the concrete. The water absorption coefficients are bigger in CDWRCer. In the coarse fraction, the absorption coefficient was 6.2% for CDWRCon and 10.7% for CDWRCer. This difference is associated with the absorption of clay materials, bricks and tiles. The fine fractions have absorption coefficients similar for both materials.

According to UNE-EN 13242 standard [38], the CDWRCon corresponds to the *AS*_0.8_ class of acid soluble sulphate. The CDWRCer presents a greater content of these parameters, relating to the declared classes (*AS_Declared_* and *S_Declared_*), with a content of 2.1 and 1.2 to acid soluble sulphate and of sulphur compounds, respectively.

The blinder used for manufacturing the concrete was a cement type BL II/B-LL 42,5 R. This cement has the following components: (i) a Clinker content comprised between 65–79%; (ii) a Limestone content of 21–35%; (iii) a Chloride content: ≤0.10; (iv) a Sulphate content: ≤4.0; and (v) a soluble toilet chromium VI content ≤ 0.0002%. It has a beginning setting of: ≥60 min and an end setting of: ≤720 min. The expansion is less than 10 mm. Resistance at 2 days: ≥20 MPa and resistance at 28 days in the interval 42.5 ≤ *R* ≤ 62.5 MPa. As an additional feature looking for the best termination, a white cement has been chosen, with a whiteness content ≥85%.

### 2.2. Mechanical Characterization of the Concrete: The 3D Digital Image Correlation Method

**Figure 4:**
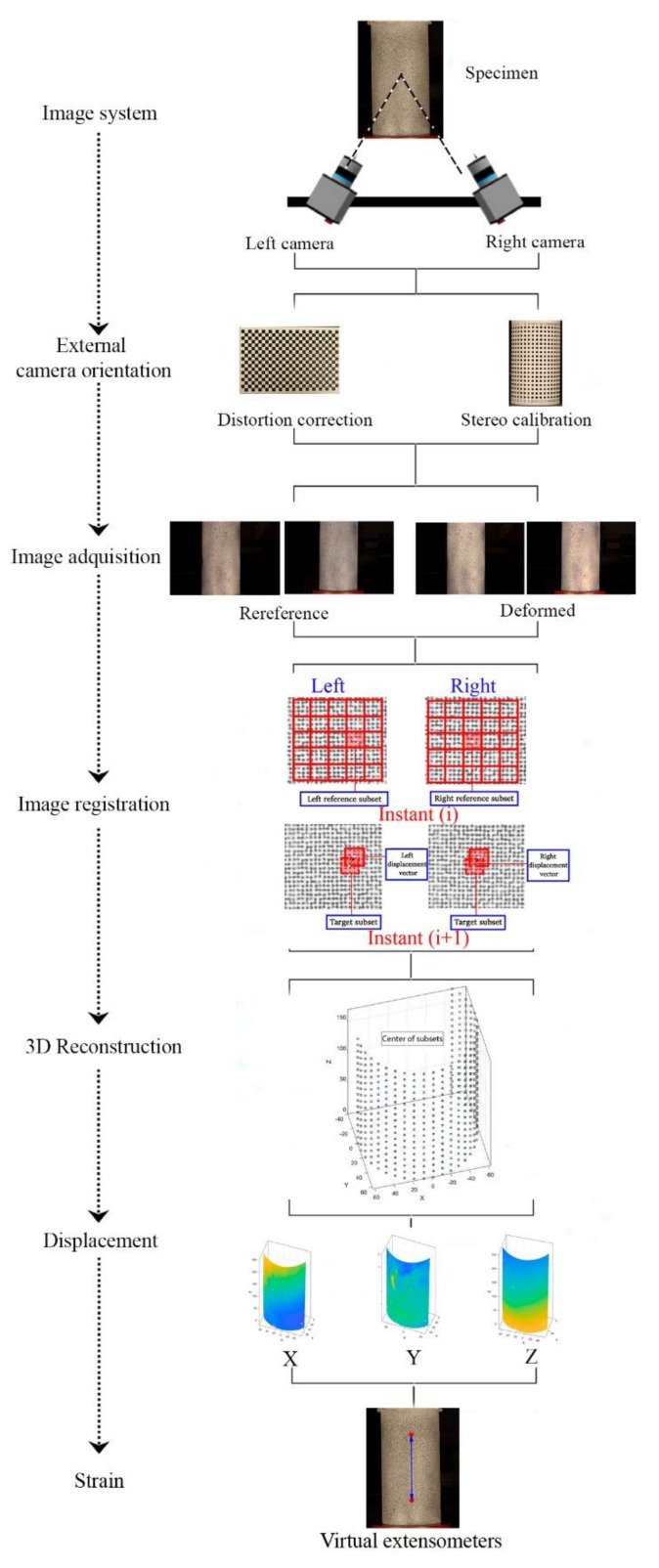
Graphical representation of the 3D-DIC approach.

This section shows the 3D digital image correlation (3D DIC) strategy used for obtaining a full field of displacements in the different concrete samples tested. Figure 4 shows the methodology followed. 

#### 2.2.1. Data Acquisition Prototype and Specimen Preparation

The concrete solutions were evaluated by means of compression tests according to guideline UNE-EN 12390-3 [48]. In order to carry out these tests, an electromechanical test machine Servosis ME-405/50/5 (Servosis, Madrid, Spain) was used with a load cell of 500 kN and the corresponding compression plates.

**Figure 5:**
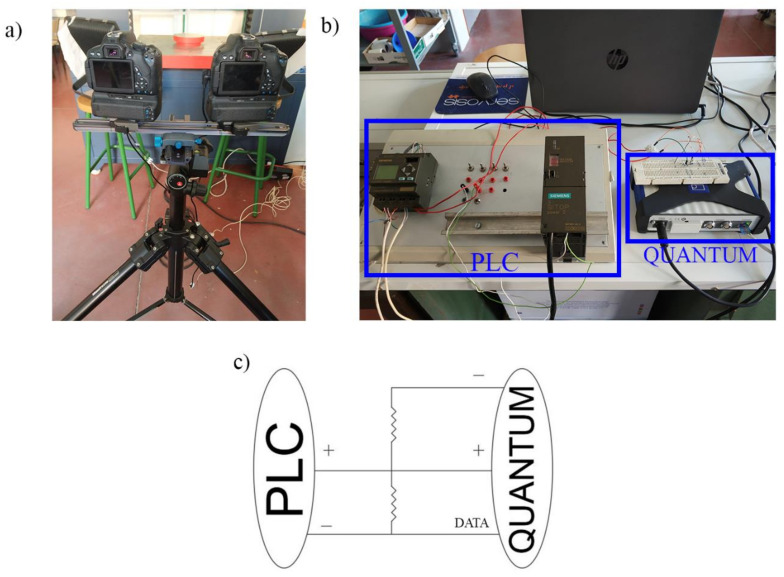
Data acquisition prototype: (**a**) platform of cameras and lighting units; (**b**) PLC and Quantum connections; and (**c**) single-line connection diagram.

In order to capture the displacements and strains suffered by the concrete solutions during compression tests, a 3D-DIC approach was used. The acquisition of these images was carried out using a low-cost 3D-DIC prototype similar to the developed by Garcia-Martin et al. [49] (Figure 5). This prototype is made up of (Table 4) (Figure 5): (i) two high resolution cameras Canon EOS 700D equipped with a 60 mm prime macro-lens; (ii) a programmable logic controller (PLC); and (iii) two neutral LED lights.

The synchronization of both cameras was carried out by means of a PLC that allowed us the programming of simultaneous shots. Furthermore, it was connected to a Quantum data acquisition platform (Figure 5b,c), allowing for the association the images to be captured with the load applied by the press.

The application of the DIC approach requires the presence of a speckle pattern that provides random intensity variations on the surface of the samples. In this sense, the aerosol technique allows us to create speckle patterns with millimetre or submillimetre spot size on the surface of the specimens [29]. The procedure to obtain this pattern consists of the following steps: (i) application of a white paint on the surface of the specimen; (ii) creation of black dots over the white surface by means of a spray; and (iii) quality evaluation according to the Mean Intensity Gradient (MIG) parameter [50].

#### 2.2.2. Camera Orientation

An orientation phase was carried out ahead of the data acquisition. This phase allowed us to pass from the 2D images to a 3D point cloud. From the present case study, the Solav et al. [51] strategy was used. This approach integrates the Bundle Adjustment (BA) algorithm with the Direct Liner Transformation (DLT) algorithm to obtain the distortion, and internal and external parameters of the cameras. 

Firstly, the inner calibration of the cameras was obtained by using the BA method. This method allowed us to minimize the overall re-projection error of the control points (corners of squares) extracted from a calibration pattern. Therefore, lens distortion can be corrected by using a non-linear distortion model, replacing the idealized coordinates with those corrected, according to a Gaussian radial distortion model (Equation (1)). In order to guarantee the accuracy and quality of this process, it is necessary to capture a set of images, generally between 50 and 100 [51], of a flat calibration target so that different positions and orientations are captured throughout the FOV.

(1) $$\left[\begin{array}{c} x_{d}\\ y_{d} \end{array}\right]\;=\;\left(1+k_{1}r^{2}+k_{2}r^{4}+k_{3}r^{6}\right)\;\cdot \;\left[\begin{array}{c} x\\ y \end{array}\right]\;+\;\left[\begin{array}{c} 2p_{1}xy\;+\;p_{2}\left(r^{2}\;+\;2x^{2}\right)\\ p_{1}\left(r^{2}\;+\;2y^{2}\right)\;+\;2p_{2}xy \end{array}\right]$$

where $r^{2}=x^{2}+y^{2}$ represents the radial distance, *r*, computed from the images’ coordinates (*x,y*); (*x_d_*, *y_d_*) are the image coordinates corrected from lens distortion; *k*_1_, *k*_2_, *k*_3_ are the radial distortion parameters; and *p*_1_, *p*_2_ are the decentering distortion parameters.

Once the lens distortion has been corrected, the DLT algorithm allows us to relate the image coordinates (*x_d_*, *y_d_*) and the object coordinates (*X’*, *Y’*, *Z’*). This algorithm provides a linear solution of 11 mathematical parameters equivalent to the non-linear model of 9 geometric parameters. The system can be solved knowing at least six points (Equation (2)). In this case a non-planar calibration object is used in which there are control points with their known 3D coordinates. Furthermore, the DLT algorithm allows us to obtain the 3D coordinates of a specific point by using the 2D coordinates of this point in at least two images.

(2) $$x_{p}=\frac{L_{1}X+L_{2}Y+L_{3}Z+L_{4}}{L_{9}X+L_{10}YL_{11}Z+1}\;y_{p}=\frac{L_{5}X+L_{6}Y+L_{7}Z+L_{8}}{L_{9}X+L_{10}YL_{11}Z+1}$$

where *x_p_* and *y_p_* are the image point coordinates and *L*_1_, *L*_2_, *L*_3_, *L*_4_, *L*_5_, *L*_6_, *L*_7_, *L*_8_, *L*_9_, *L*_10_, *L*_11_ correspond to the 11 mathematical parameters of the DLT.

#### 2.2.3. Correlation

**Figure 6:**
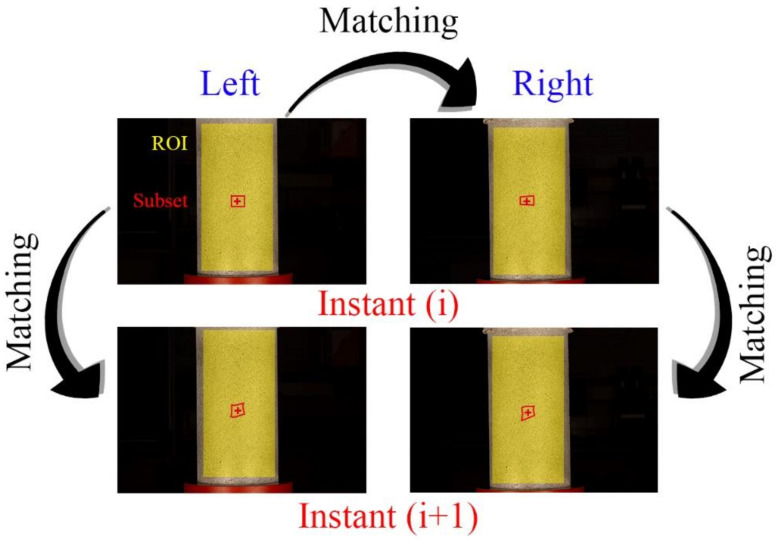
Image registration in 3D-DIC approach.

As previously stated, the proper orientation of the cameras allowed us to reconstruct the common pixels between cameras in a common 3D space. Prior to this 3D reconstruction, it was required to use a correlation algorithm that allows matching homologous pixels captured at the time *i* and its homologous at the time *i +* 1 (Figure 6) in order to obtain the displacement vector (Figure 4). 

In order to carry out the matching procedure, it is necessary to divide the Region of Interest (ROI) into smaller areas called subsets (Figure 4). The degree of similarity between the subsets of each image is evaluated using the Zero mean Normalized Cross-Correlation (ZNCC) criterion [52]. This correlation criterion is insensitive to the offset and linear scale in illumination lighting, offering the most robust noise-proof performance (Equation (3)).

(3) $$C_{ZNCC}=\frac{\sum {\bar{f}}_{i}{\bar{g}}_{i}}{\sqrt{\sum {\bar{f}}_{i}{}^{2}\sum {\bar{g}}_{i}{}^{2}}}$$

where $\bar{f}=\frac{1}{n}\;\overset{n}{\underset{i=1}{\sum }}f_{i}$, $\bar{g}=\frac{1}{n}\;\overset{n}{\underset{i=1}{\sum }}g_{i}$, ${\bar{f}}_{i}=f_{i}-\bar{f}$, ${\bar{g}}_{i}=g_{i}-\bar{g}$ with $f_{i},\;g_{i}$ representing the intensity value of the *i*th pixel point within reference subset and deformed subset, respectively.

The use of the ZNCC correlation allows for matching homologous points with pixel accuracy. In order to obtain a sub-pixel accuracy, the use of the following two-fold approach is required [53]: (i) a b-spline b-quantum interpolation scheme to pass from the discrete values of the images (0–255) to a continuous space [54]; and (ii) the Inverse Compositional Gauss-Newton method (IC-GN) for the minimization of the cost function that relates the reference subset to the deformed one. With the aim of minimizing the error accumulation due to the matching process, the Reliability-Guided Digital Image Correlation (RG-DIC) algorithm was used [55]. This algorithm starts from an initial point or seed and processes the rest of the subsets following an error minimization process.

Once the correlation process has been carried out, it is possible to perform the three-dimensional reconstruction of the corresponding points, thus obtaining a point cloud associated with the centres of the subsets placed within the ROI. To this end, the DLT parameters (*L*_1_–*L*_11_) obtained in the orientation process were used along with image coordinates (*x_p_*, *y_p_*) of the points obtained from DIC. In this way, the real coordinates of each of the points (*X*, *Y*, *Z*) can be calculated by means of Equation (4), following a least-square strategy. Since the external orientation was performed with a calibration object with known 3D coordinates, the *X*, *Y*, *Z* coordinates are obtained in the global coordinate system for all cameras.

(4) $$P={\left[\begin{array}{c} A^{T}A \end{array}\right]}^{-1}A^{T}U$$

$$A=\left[\begin{array}{c} L_{1}^{C_{R}}-L_{9}^{C_{R}}x_{p}^{C_{R}}\;\;\;\;L_{2}^{C_{R}}-L_{10}^{C_{R}}x_{p}^{C_{R}}\;\;\;\;L_{3}^{C_{R}}-L_{11}^{C_{R}}x_{p}^{C_{R}}\\ L_{5}^{C_{R}}-L_{9}^{C_{R}}y_{p}^{C_{R}}\;\;\;\;L_{6}^{C_{R}}-L_{10}^{C_{R}}y_{p}^{C_{R}}\;\;\;\;L_{7}^{C_{R}}-L_{11}^{C_{R}}y_{p}^{C_{R}}\\ L_{1}^{C_{L}}-L_{9}^{C_{L}}x_{p}^{C_{L}}\;\;\;\;sL_{2}^{C_{L}}-L_{10}^{C_{L}}x_{p}^{C_{L}}\;\;\;\;L_{3}^{C_{L}}-L_{11}^{C_{L}}x_{p}^{C_{L}}\\ L_{5}^{C_{L}}-L_{9}^{C_{L}}y_{p}^{C_{L}}\;\;\;\;L_{6}^{C_{L}}-L_{10}^{C_{L}}y_{p}^{C_{L}}\;\;\;\;L_{7}^{C_{L}}-L_{11}^{C_{L}}y_{p}^{C_{L}} \end{array}\right]$$

where:

with *C^R^* and *C^L^* corresponding to the right and left cameras, respectively.

$$P=\left[\begin{array}{c} X\\ Y\\ Z \end{array}\right]U=\left[\begin{array}{c} x_{p}^{C_{R}}-L_{4}^{C_{R}}\\ y_{p}^{C_{R}}-L_{8}^{C_{R}}\\ x_{p}^{C_{L}}-L_{4}^{C_{L}}\\ y_{p}^{C_{L}}-L_{8}^{C_{L}} \end{array}\right]$$

Once the three-dimensional coordinates of the homologous points for the entire ROI were obtained, these are used to calculate the displacements in 3D. In this way, it was possible to obtain a full field of displacements for each of the stereoscopic pairs.

### 2.3. Predictive Modelling Strategy

This section describes the strategy used for fitting both predictive models: (i) the predictive model for the unconfined compressive strength (UCS); and (ii) the predictive model for the maximum strain. As stated in the Introduction, the DIC approach is an added value in terms of the possibility of analysing the heterogeneous behaviour of the material and studying the maximum strains in the closest area to failure. This advantage allows us to establish a model to predict peak strain more precisely from the compressive strength of the concrete studied.

On the one hand, different models have been proposed for predicting the compressive strength [21,22,23] for different types and mix proportions of conventional and recycled concrete. However, the total replacement of coarse and fine aggregates represents a challenge that has not been addressed in the extensive bibliography and that aims to be solved with the predictive models proposed in this work.

Concerning the strain predictions, some design codes assumed a constant value of 0.002, meanwhile other models directly relate compressive strength with peak strain, such as the one provided by González-Fonteboa et al. [25] (Equation (5)), which has been established based on Eurocode 2 [24], considering the percentage of substitution of coarse aggregates for recycled aggregates:

(5) $$\varepsilon_{c1}\;=\;0.7\;\cdot \;{\left(f_{cm}\right)}^{0.31}\;\cdot \;\left(0.0021\;\cdot \;\%RCA+1\right)$$

where *ε_c_*_1_ is the peak strain; *f_cm_* is the compressive strength at 28 days; and *%RCA* is the percentage of replacement with recycled coarse aggregates.

However, the strain at compressive strength depends on other variables that do not take into account models, such as mix composition, shape and size of specimen or age of curing [56]. The difference in the properties of the aggregates can be decisive in the final behaviour of the recycled concretes, so there are prediction models that incorporate other variables related to the properties of the aggregates, such as the mortar content, volume, density crushing strength and shape index [23]. In this sense, the properties with the greatest influence on the final result will be studied to incorporate them into the models.

#### 2.3.1. Model Fitting

The model fitting strategy used was the Multiple Linear Regression (MLR) [57]. This fitting strategy allows us to predict the UCS and the maximum strain of the concretes by means of different inputs, such as the mix proportions and the specific characteristics of the concrete. This regression model was complemented by a variable transformation, which allows us to study the input-output relation from a non-linear perspective. 

Complementary to both regression strategies, several statistical analyses were carried out with the aim of evaluating the statistical significance of the inputs. These tests were: (i) the analysis of variance (ANOVA) test, (ii) the Levene´s test; and (iii) *t*-Student test. 

#### 2.3.2. Sensitivity Analysis

The mathematical model can be finally expressed as a relationship in which there are three inputs bounded by the experimental data, which allows us to obtain a prediction of the final output value. According to this, a good practice compromises the analysis of the influence of each input in the final output. From the present study, it was decided to carry out a sensitivity analysis based on the Monte Carlo simulation (MCS). This method allows us to generate equiprobable situations, which could be considered as suitable sampling points for a subsequent sensitivity analysis. Within this context, one of the most used strategies to carry out sensitivity analysis is the estimation of the Sobol’indices [58]. These indices assume that the variance of the model (output) can be described as a sum of the variances of the inputs (Equation (6)). The normalized version of each variance with respect to the total one allows us to obtain de Sobol’indices with different orders (from 1 to 2^n−1^) (Equation (7)). The sum of these indices is the total Sobol’ index whose value is equal to 1.

(6) $$V\left(Y\right)=\underset{i}{\sum }V_{i}+\underset{i}{\sum }\underset{j>i}{\sum }V_{ij}+\underset{i}{\sum }\underset{j>i}{\sum }\underset{k>j}{\sum }V_{ijk}+\ldots V_{123..N}$$

where *V(Y)* is the variance of the model; *V_i_ = V(E(Y|X_i_))* is the first order partial variance; *V_ij_ = V(E(Y|X_i_,X_j_))* is the second order partial variance, etc.

(7) $$S_{i}=\frac{V_{i}}{V\left(Y\right)},S_{ij}=\frac{V_{ij}}{V\left(Y\right)},$$

where *S_i_* is the first order Sobol’ index and *S_ij_* is the second-order Sobol’indices.

## 3. Experimental Results

### 3.1. Test Setup

A total of 38 tests were carried out following the guideline UNE-EN 12390-3 [48], 19 of which correspond to specimens with concrete aggregates (CDWRCon) and another 19 which correspond to specimens with ceramic aggregates (CDWRCer). For each type of concrete, four different mix proportions were used, and for each of these two different curing times were analysed (Table 5). Additionally, for dosages 2, 3 and 4, a specimen was reserved for testing with a longer curing age. It is worth mentioning the need to employ high water-cement ratios (*w*/*c*) in order to achieve good concrete workability. In addition, the variability in these values allows us to extend the validation range in modelling.

In order to optimize the configuration to be used during the 3D-DIC test, a preparation stage was carried out. The steps followed in this process were as follows: (i) application of the speckle pattern; (ii) definition of the Ground Sampling Distance (GSD), lens aperture and stereo angle; and (iii) geometric calibration and orientation of the cameras.

**Figure 7:**
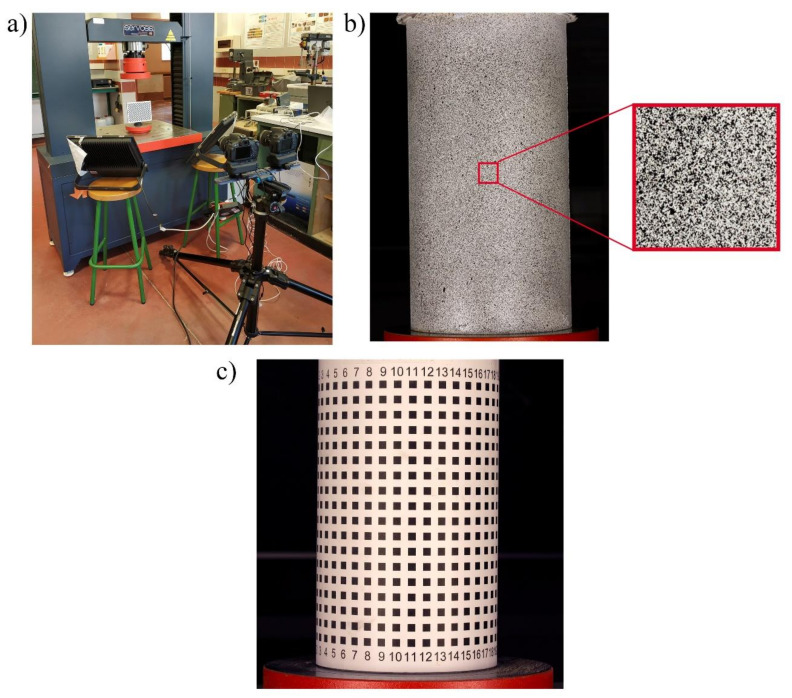
Digital Image Correlation campaign: (**a**) set-up used at the calibration stage; (**b**) detail of the speckle pattern applied; and (**c**) cylindrical calibration object.

Firstly, a speckle pattern was applied using the aerosol technique (Figure 7b). The quality of the pattern was evaluated through the covering factor [31] as well as the Mean Intensity Gradient (MIG) index [50]. For the first variable, an average value between 45–50% was obtained. Meanwhile the MIG values were comprised between 30–35, which was considered acceptable taking into account the method used [50].

The success of the 3D-DIC approach depends strongly on the GSD of the images and the stereo angle of the cameras. In this sense, an angle that is too high allows for a better precision in depth, but a lower precision in the plane, and an angle too small allows a better precision in the plane at the cost of a higher uncertainty in depth. Under this basis, the acquisition system was placed at 1.25 m with respect to the specimen (Figure 7a), achieving a GSD of 0.09 mm/px. The aperture of both lenses was established in f10, obtaining a good compromise between depth of field (which was of 180 mm) and sharpness. Additionally, a stereo angle of 10° was configured in order to avoid possible depth of field problems [59]. Taking into account that the loading speed was 0.4 MPa/s, the images were acquired each 0.6 MPa with a shutter speed of 1/100 s, capturing the first image without load in order to obtain the reference image.

Taking into account that the tests were performed on different days, the camera orientation procedure described in Section 2.2.2 was repeated for each of these days before carrying out the tests. The geometrical calibration of the camera was carried out by using a high-quality checkerboard target (Figure 7a). This target is made up of a matrix of 18×29 squares of 10 mm. Approximately 100 images were captured at different positions and angles, so that the BA algorithm allowed us to obtain the control points and calculate the lens distortion parameters, with an average re-projection error of 0.15 px for each camera.

The external orientation was carried out applying the DLT procedure, for which an image of a 125 mm diameter cylindrical object was captured (Figure 7c). The calibration object contains several control points. These points are placed on a 18 × 25 matrix with a spacing of 10 mm. It is worth mentioning that the re-projection of the control points allowed us to calculate the error associated with the reconstruction, obtaining a mean value between 0.010–0.015 mm and a Root Mean Square Error (RMSE) value between 0.155–0.179.

### 3.2. Mechanical Properties of the Concrete Evaluated

**Figure 8:**
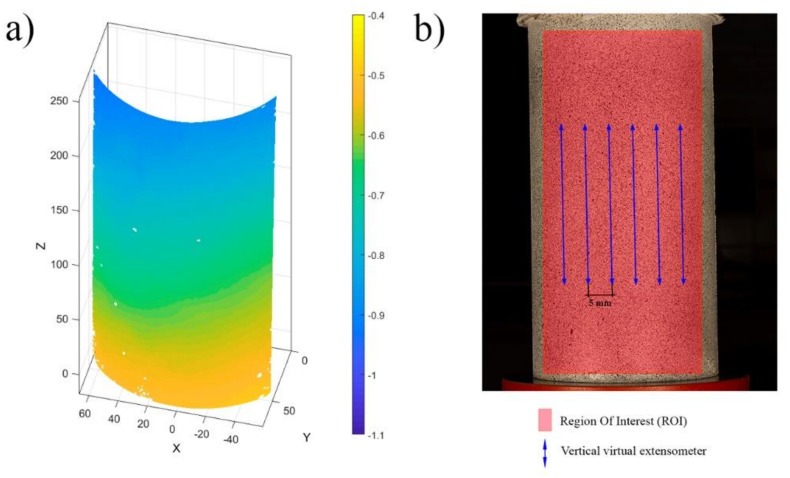
Results obtained during the 3D-DIC: (**a**) displacements obtained along the longitudinal axis; and (**b**) extraction of the maximum longitudinal strain by means of the virtual extensometer.

In order to obtain the displacement and strain on each test specimen, the 3D-DIC approach defined in Section 2.2 was carried out by using the open-source software MultiDIC [51]. This software integrates the open-source software Ncorr (Version 1.2, J. Blaber, Atlanta, GA, USA) [53]. Regarding DIC parameters, a subset size of 20 × 20 pixels and a 65% overlap (step of 7 pixels) were considered to ensure a proper DIC configuration [29]. The interpolation between points was carried out by considering a linear shape function. Finally, the 3D reconstruction of the sample was obtained by applying the DLT algorithm, allowing us to obtain a full field of displacements (Figure 8a).

The strains suffered by the specimen were captured at different locations. To this end the following strategy was used: (i) creation of several virtual extensometers to evaluate the longitudinal strains (Figure 8b); (ii) extraction the peak longitudinal strain in the state of load prior to failure; and (iii) selection of the virtual extensometer with the maximum peak strain, corresponding to the failure zone.

With the aim of obtaining a wide population, 22 virtual extensometers were placed in each specimen with a separation of 5 mm. In this sense, it is worth mentioning the high differences found between the values of the different virtual extensometers, which indicate the high heterogeneous behaviour of these materials. This heterogeneity can be seen in the high CoV corresponding to the virtual extensometers of some sample specimens shown in the Table 6. In order to establish a more precise model to predict strains, the virtual extensometers were analysed and those that corresponded to the failure zone were selected, such as peak strain.

## 4. Strength and Strain Models

### 4.1. Concrete Strength Model

The concrete strength model was obtained by using the MLR approach. This analysis was carried out with the assistance of the IBM SPSS Statistics software (Version 26.0, IBM Corp., Armonk, NY, USA). The input variables considered during this stage were: (i) the time (*t*); (ii) the water–cement ratio (*w*/*c*); and (iii) the percentage of material belonging to the class *Rc* + *Ru* (*RcRu*) established by UNE-EN 13242 standard [38], where *Rc* corresponds to concrete, concrete products, mortar and concrete masonry parts, and *Ru* correspond to untreated aggregates and natural stone aggregates treated with hydraulic binders.

**Figure 9:**
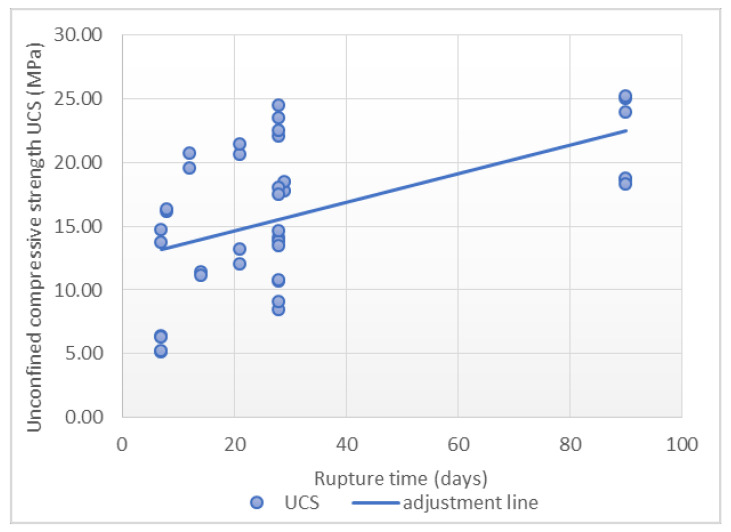
Relationship between rupture time and unconfined compressive strength.

**Figure 10:**
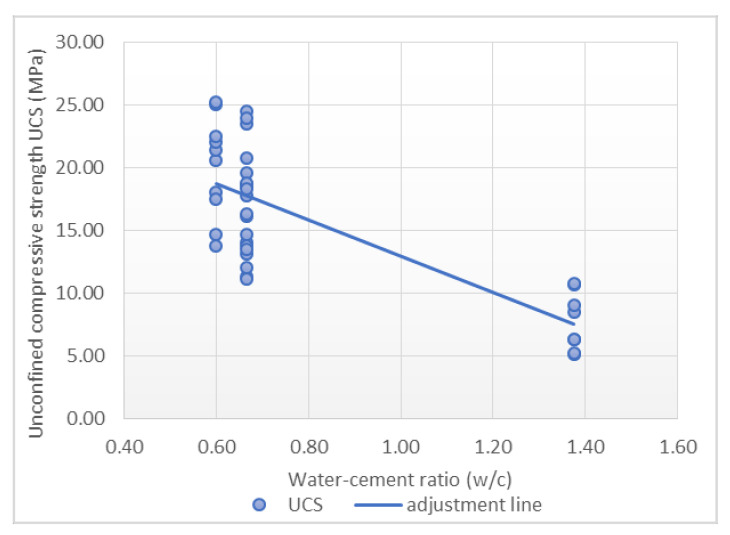
Relationship between water-cement ratio and unconfined compressive strength.

**Figure 11:**
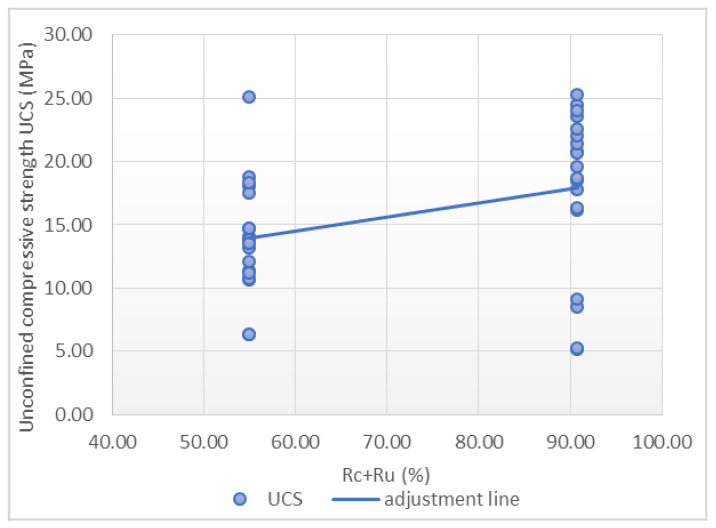
Relationship between *Rc* + *Ru* and unconfined compressive strength.

A total of 38 points were used to carry out the adjustment. Scatterplots with a trend line are showed in Figure 9, Figure 10 and Figure 11, which express the correlation between each independent variable and the compressive strength. A positive correlation between the rupture time and *Rc + Ru* with the maximum strength was observed. The water-cement ratio shows an inverse correlation with respect to the maximum strength. 

It is worth mentioning the low correlation between the individual inputs considered and the final output. Furthermore, in Figure 9, Figure 10 and Figure 11 it can be seen how outliers can generate a correlation and a linear relationship between the variables that does not exist, according to the fourth case of Anscombe’s quartet. As a result, a multilinear regression model was applied in order to consider all these variables in the same prediction model. In order to improve this regression, a transformation of the variables was carried out. In this case, the most satisfactory results correspond to the use of UCS as a cubic root and rupture time as a fifth root. The adjustment with the transformed variables resulted in a Pearson’s correlation coefficient value of 0.921, representing a high correlation, and the determination coefficient is 0.848, with a standard error of 0.137, as is shown in Table 7.

Table 8 shows the results obtained after applying the ANOVA strategy as well as Levene´s test. This test revealed a homoscedasticity with an F = 63.009 and a significance of 0.000 for 37 degrees of freedom. In this case, variances are significantly different and factors such as the rupture time, water-cement ratio and *Rc + Ru* have a statistical significance with the UCS. 

The increase in the quality of the adjustment with the transformed variables, based on the value of the correlation coefficient, was not significantly high. However, it is considered advantageous to use the transformations in order to achieve the best possible approximation. 

The obtained coefficients and the Student’s t-test results are shown in Table 9 with a high value of the *t* statistic, between 4.211–10.069. All the variables are significant (sig. < 0.050).

Therefore, the equation that represents the best fit is Equation (8).

(8) $$\sqrt[{3}]{\mathrm{UCS}}=0.385\times \sqrt[{5}]{t}-0.774\times w/c+0.005\times RcRu+1.983$$

where, UCS is unconfined compressive strength (MPa), *t* is rupture time (days), *w/c* is water-cement ratio and *RcRu* is material belonging to the class *Rc + Ru*. There is a validity range to the significance of the model that is shown in Table 9. UCS has a range between 5.13 and 25.27 MPa. Rupture time is from 7 until 90 days. The water–cement ratio is from 0.60 until 1.38 and *Rc + Ru* is between 54.99 and 90.74. 

In order to understand which variables are the most relevant in the strength prediction model of the recycled concrete, a Sobol sensitivity analysis was carried out. A total of 500,000 simulations were performed in order to carry out this analysis through a Monte Carlo Simulation. For each of the simulations, the input variables were modified within the ranges obtained from the experimental results.

**Figure 12:**
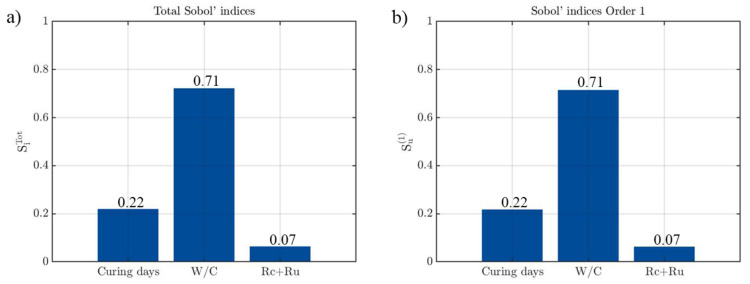
Results obtained during the sensitivity analysis of the strength model: (**a**) Total Sobol’indices and; (**b**) First-order Sobol’indices.

Figure 12 indicates that the water/cement ratio is the most relevant parameter in the compressive strength prediction model. This property explains 71% of the total variance, agreeing with the Student’s t-coefficients previously analysed. The rupture time has a greater influence in the final output with a Sobol index of 0.22 (22%), while *Rc + Ru* percentage is the variable that least affects the prediction. The great similarity between the first and the total Sobol’indices highlights the absence of a second-order effect.

### 4.2. Concrete Strain Model

**Figure 13:**
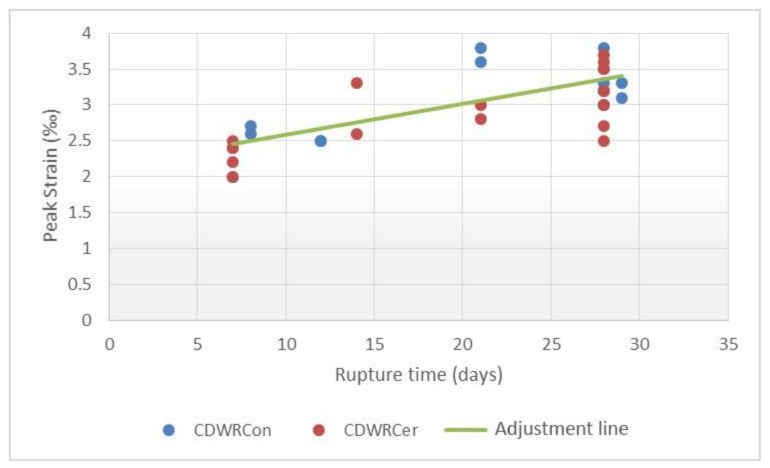
Relationship between rupture time and peak strain.

**Figure 14:**
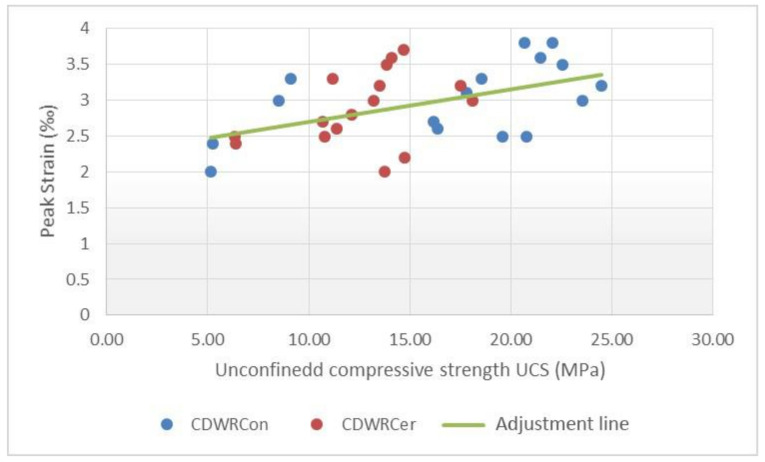
Relationship between unconfined compressive strength and peak strain.

**Figure 15:**
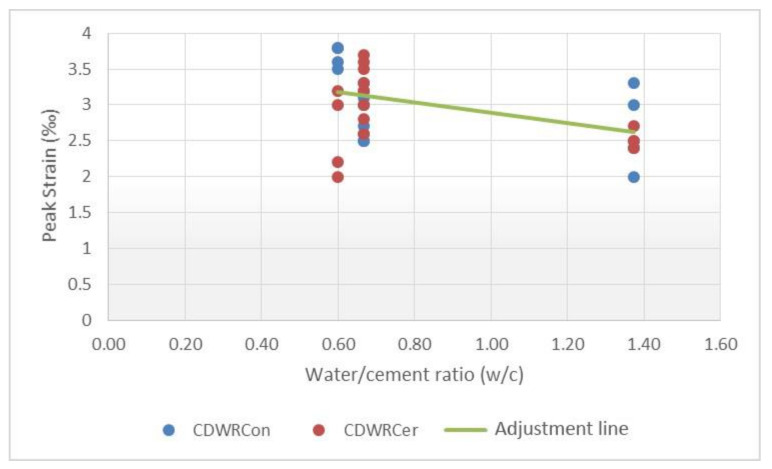
Relationship between water-cement ratio and peak strain.

The inputs evaluated for predicting the maximum strain of the concretes were the compressive strength, the curing age and the water–cement ratio. Scatterplots with a linear trend are shown in Figure 13, Figure 14 and Figure 15, which express the correlation between each independent variable and the peak strain.

The results in this case, for the simple regressions, also show a low correlation and outliers in Figure 15. Taking into consideration the values previously obtained, several multiple linear regressions with variable transformation were carried out. It is worth mentioning that a total of two maximum strain equations were obtained due to the dissimilarity of the data for the CDWRCon and CDWRCer. For this adjustment, a total of 32 samples were used.

The following transformations were used during the adjustment: (i) a square root for the peak strain; (ii) a cubic root for the UCS; and (iii) a fifth root for the rupture time. As a result of the transformations carried out, it was possible to obtain an adjustment for the concrete manufactured with CDWRCon with a Pearson’s correlation coefficient value of 0.859, with the determination coefficient as 0.738 and a standard error of 0.090 (Table 10).

The analysis of variance (ANOVA) (Table 11) showed that the variables have variances significantly different and the predictors have a statistical significance on the peak strain prediction. The result in the Levene’s test yielded a F = 11.272 with 0.001 of significance for 15 degrees of freedom.

The obtained coefficients and the Student’s t-test results are shown in Table 12. They show low values, especially for the UCS and *w*/*c* ratio. The results show that these variables are not significant (sig. > 0.050). These values indicate the presence of anomalies that prevent obtaining an accurate prediction model for this type of concrete.

In order to determine what happens in the CDWRCon model, the data used to calculate the model were analysed individually and compared with the CDWRCer data. A greater data dispersion was found for the concretes made up by concrete waste, while ceramics have a more uniform distribution with no empty ranges (Figure 13). This issue could be attributed to an insufficient population. Thus, although the R^2^ coefficient can be considered as acceptable, the parameters are not correct and the predictions for values of the inputs differ from those used to generate the model, which may result in erroneous predictions. 

On the other hand, the model for the concrete manufactured with CDWRCer yielded a better adjustment with a Pearson’s correlation coefficient value of 0.917, while the determination coefficient is 0.840 with a standard error of 0.085 (Table 13).

The analysis of variance (ANOVA) (Table 14) is similar to the previous case, with a result in the Levene’s test of F = 11.402 with 0.001 of significance for 15 degrees of freedom. This analysis shows that the variables have variances significantly different and the predictors have a statistical significance on the peak strain prediction. 

The Student’s t-test results (Table 15) show a good value of the *t* statistic, between 2.442–5.036. These results indicate that all the variables are significant (sig. < 0.050) and the model seems to have a correct behaviour in this case.

Therefore, the peak strain prediction model can be represented by the Equation (9).

(9) $$\sqrt{\varepsilon_{p}}\;=\;1.963+0.740\;\cdot \;\sqrt[{5}]{t}-0.550\;\cdot \;\sqrt[{3}]{\mathrm{UCS}}-0.386\;\cdot \;w/c$$

where $\varepsilon_{p}$ is the peak strain (‰); *t* is rupture time (days); UCS represents the unconfined compressive strength (MPa); and *w*/*c* is the water-cement ratio.

Further, the data obtained from the strain prediction model were subjected to a validation analysis. Thus, the predictions for the experimental data were simulated and compared with the results obtained during the campaign. A mean discrepancy of 6.1% was obtained for the CDWRCer model. These results were considered acceptable taking into account the low peak strain values as well as the precision required in this type of prediction.

In order to understand which variables are the most relevant in the strain prediction model of the recycled concrete, a Sobol sensitivity analysis was carried out. A total of 500,000 simulations were performed to carry out this analysis through a Monte Carlo Simulation. For each of the simulations, the input variables were modified within the ranges obtained from the experimental results (curing days [7–30 days]; strength [5–25 MPa]; and water-cement ratio [0.60–1.38]).

**Figure 16:**
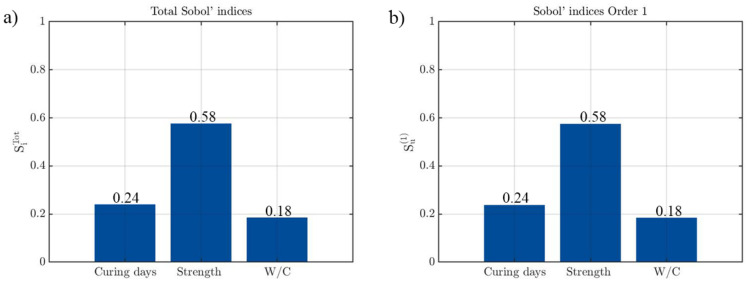
Results obtained during the sensitivity analysis of CDWRCer: (**a**) Total Sobol’indices and; (**b**) First-order Sobol’indices.

Figure 16 indicates that in the case of CDWRCer, the compressive strength of the concrete is the most relevant parameter in the peak strain prediction model. This mechanical property explains 58% of the total variance, which agrees with other studies in which it is stated that strength is the main variable in this type of model [23,56]. The other two input parameters have a similar influence in the results, 24% corresponding to the curing age and 18% corresponding to water-cement ratio. The great similarity between the first and the total Sobol’indices highlights the absence of a second-order effect.

## 5. Conclusions

This work aimed at investigating a predictive model for the determination of the UCS and the maximum strain in non-structural concretes made up by two types of recycled waste: concrete and ceramic wastes. The inputs required for fitting these models have been obtained by means of an extensive experimental campaign, which include granulometric analysis, physical and chemical analysis, and compression test among others. It is worth mentioning the use of the 3D-DIC as a remote sensing approach able to obtain a full-field of strains. This property allows us to accurately determine the peak strain of the concrete, which showed a high heterogeneity depending on the area considered. 

Within the predictive model strategy, the simple regressions yielded low correlation values for the individual variables, so finally an MLR model was adjusted and showed that there is a good correlation between all the variables considered together. In addition, the transformations of variables made it possible to minimize errors. This highlights the need to incorporate different variables to obtain a correct predictive model.

On the one hand, the adjustment obtained using MLR demonstrates that the variables’ rupture time, water-cement ratio and *Rc* + *Ru* are able to predict the UCS with a determination coefficient of 0.848 within the validity range, with a standard error of 0.137. The coefficients showed that the *w*/*c* ratio has the greatest influence on compressive strength. 

On the other hand, the strain prediction model allows for estimating the peak strain as a function of three input variables: rupture time, unconfined compressive strength and water/cement ratio. The sensibility analysis showed that the UCS has the greatest influence on peak strain. Thus, the strength value obtained from the previous model can be employed to estimate the peak strain.

The results obtained for the concrete manufactured with ceramic waste can be considered satisfactory, since the R^2^ coefficient of 0.840 is supported by several statistical analyses that verified the statistical significance of the inputs, as well as the low discrepancy in the verification with the experimental data. However, the results obtained for the concrete manufactured with concrete waste model show more anomalous values with a low R^2^ coefficient and less satisfactory results during statistical analysis. These results show the complexity of establishing a prediction model for this type of concrete, making a larger population necessary to carry out the adjustment, which will try to be integrated into future research.

One of the main future works will focus on scaling up the data from experimental campaigns in order to achieve a database that allows us to contrast and scale the results of the modelling and check the error. Along with this, one of the main interests focuses on the use of the full-field data provided by the 3D-DIC approach to evaluate other properties that could be of great interest in this type of concrete, such as elasticity, shrinkage or behaviour at the aggregate-cement interface. For this purpose, other types of tests, such as bending tests, could give rise to greater possibilities regarding the analysis of these properties under a 3D-DIC approach. Additionally, further dosages will be carried out in order to improve the influence of the *w*/*c* in the compressive strength and maximum strain. To this end, the use of superplasticizers will be planned.

Additionally, the proposed methodology will be implemented in concrete with other types of recycled aggregates that allow for higher performance for structural uses and thus be possible to carry out numerical simulations with the properties obtained.

## Figures and Tables

**Table 1:** Proportions of the different aggregates.

Components	CDWRCon		CDWRCer	
	Contents (%)	Categories	Contents (%)	Categories
*Rc*	82.5	*Rc*_80_	42.4	*Rc_Declared_*
*Rc + Ru*	90.7	*Rcu*_90_	55.0	*Rcu*_50_
*Rb*	0.8	*Rb*_10−_	39.6	*Rb*_50−_
*Ra*	8.3	*Ra*_10−_	-	-
*Rg*	-	-	0.1	*Rg*_2−_
*X*	0.1	*X*_1−_	5.2	-
*FL*	≤0.1	*FL*_5−_	≤0.1	*FL*_5−_

**Table 2:** Aggregate type and particle size distribution characteristics of the CDWRCon and CDWRCer.

Material	*D*_10_ (mm)	*D*_30_ (mm)	*D*_50_ (mm)	*D*_60_ (mm)	*C_u_*	*C_c_*	% Fines	% Sand Size	% (4.75–9.5) mm	% (9.5–40.0) mm
CDWRCon	0.08	1.0	2.5	6.0	75.0	2.1	11.1	56.1	1.2	32.0
CDWRCer	0.08	1.2	7.0	11.0	137.5	1.6	10.4	43.5	12.9	38.6

**Table 3:** Physical and chemical parameters for CDWRCon and CDWRCer.

Material	*SE*_4_	*LA*	*OM*	*SS*	*WA_c_* (%)	*WA_f_* (%)
CDWRCon	55.9	43.0	0.14	1.1	6.2	4.0
CDWRCer	45.3	52.0	-	-	10.7	4.3

**Table 4:** Canon 700D with macrolens technical specifications.

Sensor Type	CMOS APS-C
Sensor size	22.3 × 14.9 mm^2^
Crop factor	1.61
Pixel size	4.3 μm
Image size	5184 × 3456 px
Total pixels	18.5 Mpx
Focal length	60 mm
Closer focused distance	254 mm
Lens magnification	1:1 (life size)
Dimensions	133.1 × 99.8 × 78.8 mm

**Table 5:** Typology and characteristics of the tested specimens.

Dosage	Specimen	*w*/*c* Ratio	Curing Days
CDWRCon 1	CDWRCon 1-1	1.38	7
	CDWRCon 1-2		7
	CDWRCon 1-3		28
	CDWRCon 1-4		28
CDWRCer 1	CDWRCer 1-1	1.38	7
	CDWRCer 1-2		7
	CDWRCer 1-3		28
	CDWRCer 1-4		28
CDWRCon 2	CDWRCon 2-1	0.67	8
	CDWRCon 2-2		8
	CDWRCon 2-3		29
	CDWRCon 2-4		29
	CDWRCon 2-5		90
CDWRCer 2	CDWRCer 2-1	0.60	7
	CDWRCer 2-2		7
	CDWRCer 2-3		28
	CDWRCer 2-4		28
	CDWRCer 2-5		90
CDWRCon 3	CDWRCon 3-1	0.67	12
	CDWRCon 3-2		12
	CDWRCon 3-3		28
	CDWRCon 3-4		28
	CDWRCon 3-4		90
CDWRCer 3	CDWRCer 3-1	0.67	14
	CDWRCer 3-2		14
	CDWRCer 3-3		28
	CDWRCer 3-4		28
	CDWRCer 3-5		90
CDWRCon 4	CDWRCon 4-1	0.60	21
	CDWRCon 4-2		21
	CDWRCon 4-3		28
	CDWRCon 4-4		28
	CDWRCon 4-5		90
CDWRCer 4	CDWRCer 4-1	0.67	21
	CDWRCer 4-2		21
	CDWRCer 4-3		28
	CDWRCer 4-4		28
	CDWRCer 4-5		90

**Table 6:** Results obtained from the mechanical characterization of some specimens analysed by the 3D-DIC approach.

Specimen	Mean	Lower Bound	Upper Bound	CoV (%)
CDWRCon 1-1	0.0017	0.0014	0.0020	9.90
CDWRCon 2-3	0.0025	0.0022	0.0031	7.19
CDWRCon 3-2	0.0020	0.0018	0.0025	10.16
CDWRCer 1-1	0.0019	0.0013	0.0024	16.76
CDWRCer 2-3	0.0028	0.0025	0.0030	5.44
CDWRCer 3-2	0.0024	0.0020	0.0026	6.37

**Table 7:** Determination coefficients for UCS model.

Summary Model			
*R*	*R* ^2^	Adjusted *R*^2^	Standard Error
0.921 ^a^	0.848	0.834	0.137

^a^ predictors: constant, $\sqrt[{5}]{t}$ (days), *w/c*, *Rc + Ru* (%).

**Table 8:** Analysis of variance for UCS model.

ANOVA ^a^					
Model	Squares Sum	Degrees of Freedom	Average	F	sig.
regression	3.531	3	1.177	63.009	0.000 ^b^
remainder	0.635	34	0.019		
Total	4.166	37			

^a^ dependent variable: $\sqrt[{3}]{\mathrm{UCS}}\;$(MPa); ^b^ predictors: constant, $\sqrt[{5}]{t\;}$(days), *w/c*, *Rc + Ru* (%).

**Table 9:** Determination coefficients for UCS model. Dependent variable: $\sqrt[{3}]{\mathrm{UCS}}$.

Model	No Standard Coefficients		Standard Coefficients	*t*	sig.
	*B*	Standard Error	*β*		
Constant	1.983	0.197		10.069	0.000
*t* (days)	0.385	0.076	0.354	5.040	0.000
*w*/*c*	−0.774	0.077	−0.702	−9.991	0.000
*RcRu* (%)	0.005	0.001	0.282	4.211	0.000

**Table 10:** Determination coefficients for strain model of CDWRCon.

Summary Model			
*R*	*R* ^2^	Adjusted *R*^2^	Standard Error
0.859 ^a^	0.738	0.673	0.090

^a^ predictors: constant, $\sqrt[{5}]{t}$ (days), $\sqrt[{3}]{\mathrm{UCS}}$ (MPa), *w*/*c.*

**Table 11:** Analysis of variance for strain model of CDWRCon.

ANOVA ^a^					
Model	Squares Sum	Degrees of Freedom	Average	F	sig.
regression	0.271	3	0.090	11.272	0.001 ^b^
remainder	0.096	12	0.008		
Total	0.368	15			

^a^ dependent variable: $\sqrt{\varepsilon_{p}}\;$(‰); ^b^ predictors: constant, $\sqrt[{5}]{t}$ (days), $\sqrt[{3}]{\mathrm{UCS}}$ (MPa), *w*/*c.*

**Table 12:** Determination coefficients for strain model of CDWRCon. Dependent variable: $\sqrt{\varepsilon_{p}}$.

Model	No Standard Coefficients		Standard Coefficients	*t*	sig.
	*B*	Standard Error	*β*		
Constant	1.323	0.715		1.849	0.089
*t* (days)	0.692	0.181	0.885	3.816	0.002
UCS (MPa)	−0.213	0.271	−0.537	−0.787	0.447
*w*/*c*	−0.353	0.296	−0.740	−1.192	0.256

**Table 13:** Determination coefficients for strain model of CDWRCer.

Summary Model			
*R*	*R* ^2^	Adjusted *R*^2^	Standard Error
0.917 ^a^	0.840	0.775	0.085

^a^ predictors: constant, $\sqrt[{5}]{t}$ (days), $\sqrt[{3}]{\mathrm{UCS}}$ (MPa), *w*/*c.*

**Table 14:** Analysis of variance for strain model of CDWRCer.

ANOVA ^a^					
Model	Squares Sum	Degrees of Freedom	Average	F	sig.
regression	0.244	3	0.081	11.402	0.001 ^b^
remainder	0.086	12	0.007		
Total	0.330	15			

^a^ dependent variable: $\sqrt{\varepsilon_{p}}\;$(‰); ^b^ predictors: constant, $\sqrt[{5}]{t}$ (days), $\sqrt[{3}]{\mathrm{UCS}}$ (MPa), *w*/*c.*

**Table 15:** Determination coefficients for strain model of CDWRCer. Dependent variable: $\sqrt{\varepsilon_{p}}$.

Model	No Standard Coefficients		Standard Coefficients	*t*	sig.
	*B*	Standard Error	*β*		
Constant	1.963	0.485		4.044	0.002
*t* (days)	0.740	0.147	1.010	5.036	0.000
UCS (MPa)	−0.550	0.225	−0.803	−2.442	0.031
*w*/*c*	−0.386	0.127	−0.853	−3.030	0.010

## Data Availability

Data sharing is not applicable.
